# Cerebral Amyloid-β Deposition, Axial Features, and Cognitive Alterations in Patients with Parkinson’s Disease Treated with Bilateral STN-DBS: A Long-Term Cohort Study

**DOI:** 10.3390/jpm14121150

**Published:** 2024-12-10

**Authors:** Francesco Cavallieri, Alessandro Fraternali, Annachiara Arnone, Isabella Campanini, Alessandro Marti, Annalisa Gessani, Valentina Fioravanti, Maria Angela Molinari, Giulia Di Rauso, Francesca Antonelli, Vittorio Rispoli, Alberto Feletti, Riccardo Stanzani, Benedetta Damiano, Sara Scaltriti, Lorenzo Cavazzuti, Elisa Bardi, Maria Giulia Corni, Francesca Cavalleri, Giuseppe Biagini, Giacomo Pavesi, Mirco Lusuardi, Carla Budriesi, Andrea Merlo, Annibale Versari, Franco Valzania

**Affiliations:** 1 Neurology Unit, Neuromotor & Rehabilitation Department, Azienda USL-IRCCS di Reggio Emilia, 42123 Reggio Emilia, Italy; francesco.cavallieri@ausl.re.it (F.C.); alessandro.marti@ausl.re.it (A.M.); valentina.fioravanti@ausl.re.it (V.F.); giulia.dirauso@ausl.re.it (G.D.R.); franco.valzania@ausl.re.it (F.V.); 2 Nuclear Medicine Unit, Azienda USL-IRCCS di Reggio Emilia, 42123 Reggio Emilia, Italy; annachiara.arnone@ausl.re.it (A.A.); annibale.versari@ausl.re.it (A.V.); 3 LAM-Motion Analysis Laboratory, Department of Neuromotor and Rehabilitation, Azienda USL-IRCCS di Reggio Emilia, S. Sebastiano Hospital, 42015 Correggio, Italy; isabella.campanini@ausl.re.it (I.C.); benedetta.damiano@ausl.re.it (B.D.); sara.scaltriti@ausl.re.it (S.S.); lorenzo.cavazzuti@ausl.re.it (L.C.); andrea.merlo@ausl.re.it (A.M.); 4 Neurology Unit, Department of Neuroscience, S. Agostino Estense Hospital, Azienda Ospedaliero-Universitaria di Modena, 41126 Modena, Italy; annalisa92.gessani@gmail.com (A.G.); m.molinari@ausl.mo.it (M.A.M.); antonelli.f@gmail.com (F.A.); vit.rispoli@gmail.com (V.R.); elisa.bardi7@gmail.com (E.B.); mariagiulia.corni@gmail.com (M.G.C.); budriesic@gmail.com (C.B.); 5 Clinical and Experimental Medicine PhD Program, University of Modena and Reggio Emilia, 41125 Modena, Italy; 6 Department of Neurosciences, Biomedicine, and Movement Sciences, Institute of Neurosurgery, University of Verona, 37126 Verona, Italy; alberto.feletti@gmail.com; 7 Neurosurgery Unit, Azienda Ospedaliero-Universitaria of Modena, Ospedale Civile Baggiovara (OCB) Hospital, 41126 Modena, Italy; stanzani.riccardo@aou.mo.it (R.S.); pavesi.giacomo@aou.mo.it (G.P.); 8 Division of Neuroradiology, Department of Neuroscience, Nuovo Ospedale Civile S. Agostino Estense, 41126 Modena, Italy; fcavalleri@hotmail.com; 9 Department of Biomedical, Metabolic and Neural Sciences, University of Modena and Reggio Emilia, 41125 Modena, Italy; gbiagini@unimore.it; 10 Neuromotor & Rehabilitation Department, Azienda USL-IRCCS di Reggio Emilia, 42123 Reggio Emilia, Italy; mirco.lusuardi@ausl.re.it

**Keywords:** amyloid-β, axial, cognitive, deep brain stimulation, flutemetamol, Parkinson’s disease, STN-DBS

## Abstract

**Objectives:** Our aim was to evaluate the possible long-term cerebral deposition of amyloid-β in patients with PD treated with subthalamic nucleus deep brain stimulation (STN-DBS) and its possible influence on axial and cognitive variables. **Methods:** Consecutive PD patients treated with bilateral STN-DBS with a long-term follow-up were included. The amyloid-β deposition was evaluated postoperatively through an 18F-flutemetamol positron emission tomography (PET) study. Axial symptoms were assessed using a standardized clinical–instrumental approach. The speech was assessed by perceptual and acoustic analysis, while gait was assessed by means of the instrumented Timed Up and Go test (iTUG). Motor severity was evaluated by applying the UPDRS part III score and subscores, while cognitive functions were assessed through a complete neuropsychological assessment. Different stimulation and drug conditions were assessed: on-stimulation/off-medication, off-stimulation/off-medication, and on-stimulation/on-medication conditions (single- and dual-task). **Results:** In total, 19 PD patients (male: 11; age: 63.52 years; on-stimulation/on-medication UPDRS-III: 17.05) with a five-year postoperative follow-up were included. The amyloid-β deposition was found in 21% of patients (4/19) with a prevalent involvement of prefrontal, limbic, and parietal areas. Compared with patients without amyloid-β deposition, PD patients with positive 18F-flutemetamol in the PET study showed a higher preoperative UPDRS-I (*p* = 0.037) score. **Conclusions:** Our results suggest that in the long term, after STN-DBS, a significant percentage of PD patients may present brain amyloid-β deposition. However, larger samples are needed to evaluate the possible role of amyloid-β deposition in the development of axial and cognitive alterations after surgery.

## 1. Introduction

Subthalamic nucleus deep brain stimulation (STN-DBS) represents a short- and long-term effective treatment in advanced Parkinson’s disease (PD) [1]. STN-DBS allows for a stable improvement in motor complications, tremor, and rigidity with a less relevant effect on axial symptoms (i.e., gait and balance symptoms, speech and swallowing troubles) and cognitive decline, which are the main causes of long-term disability [2,3]. Other possible targets for neurostimulation in PD include the ventral intermediate nucleus (VIM) of the thalamus and the Globus Pallidus Internus, but, to date, the STN is the most widely used target globally [4,5]. Many studies have analyzed axial symptoms in PD patients with an instrumental approach focusing only on gait and postural alterations or speech disturbances. The very few studies that have instrumentally assessed the whole spectrum of axial symptoms in PD have shown the presence of similarities between spatial-temporal gait and speech parameters [6]. Anatomopathological data have confirmed that the neurodegeneration of central dopaminergic pathways considered the hallmark of PD, is accompanied by the contemporary involvement of other neurotransmitter pathways (i.e., cholinergic, serotoninergic) [7,8,9]. The prevalent involvement of the cholinergic system could be associated with a clinical “cholinergic” phenotype dominated by axial symptoms, cognitive deterioration, and cerebral amyloid-β (Aβ) deposition [10,11]. Previous studies have reported a 20–30% prevalence of positive amyloid PET scans in patients with PD without dementia and MCI-PD patients [12,13], underlying the possible co-pathology in these patients. Based on these premises, the aim of the study is to evaluate the possible long-term cerebral deposition of amyloid-β in a cohort of PD patients treated with STN-DBS and its possible influence on axial and cognitive variables.

## 2. Materials and Methods

### 2.1. Participants

In this longitudinal cohort study, we enrolled PD patients treated consecutively with bilateral STN-DBS from 2012 to 2017. A previous diagnosis of PD was made in accordance with the UK Brain Bank criteria [14]. Moreover, at the time of surgery, all patients suffered from disabling motor complications. Exclusion criteria included the following factors: a history of ischemic or hemorrhagic stroke after surgery; a history of complications related to STN-DBS surgery, which led to neurological deficits; and head trauma or other focused brain injuries appearing during the postoperative follow-up. The study (BASCOSTIM-PD) was approved by the local ethics committee (protocol number: 2019/0056629), and written informed consent was obtained from participants according to the Declaration of Helsinki [15].

### 2.2. Clinical Assessment

All patients underwent neurological evaluation, instrumental analysis of gait, and an acoustic–perceptual assessment of speech on the same day. In particular, we assessed different conditions, including the following: on-stimulation/off-medication (12 h washout of dopaminergic drugs); off-stimulation/off-medication (stimulation turned off for at least 1 h); and on-stimulation/on-medication (stimulation was turned on, and dopaminergic therapy was administered [early morning levodopa equivalent daily dose (LEDD) plus 30%]). The Unified Parkinson’s Disease Rating Scale (UPDRS) part III and the Hoehn and Yahr (H&Y) stage were administered to quantify disease severity. Furthermore, we calculated the following subscores: postural instability/gait disorders (PIGDs), tremor, and akinesia subscores [3,16,17]. Based on these subscores, we also calculated the PD motor phenotype (PIGD, indeterminate or tremor dominant [TD]) [18]. A detailed neuropsychological assessment was also performed both before surgery and at long-term evaluation. Several items were included, such as spatial perception (localization of numbers), Raven’s progressive matrices, the Stroop test and trail-making test part B, and phonemic fluency [19]. In addition, all patients were classified as PD without dementia, PD–mild cognitive impairment (PD-MCI), and dementia associated with PD (PDD) based on current criteria [20,21]. Screening for the presence of mutations in the α-synuclein, Parkin, glucocerebrosidase-1 (GBA1), and leucine-rich repeat kinase-2 genes [22,23] was also performed in all patients. The total amount of dopaminergic medications per day was calculated as the levodopa equivalent daily dose (LEDD) mg [24].

### 2.3. Gait Instrumental Analysis

Gait instrumental analysis was performed by applying a wearable inertial sensor secured with an elastic belt at the sacrum level (S1). We used the commercial device G-WALK (BTS Bioengineering^TM^, sampling frequency 100 Hz), which provides the 3D linear acceleration, angular velocity, and magnetic field vector. The instrumented Timed Up and Go test (iTUG) [25] was performed in the different stimulation and medication conditions reported above. In each condition, the patient was asked to perform the test three consecutive times to obtain a total of twelve acquisitions. The iTUG test was performed using the standard test procedure [26] and videotaped. Data processing has been previously published [3,16]. The following variables were then extracted: the overall duration of the iTUG; the duration of each phase; the root mean square (RMS) of trunk acceleration for each phase; and the maximum speed of rotation of the trunk about the cranio-caudal axis (which is related to the subject’s ability to rotate quickly).

### 2.4. Speech Assessment

The speech assessment was performed in a silent environment, applying a digital device kept 20 cm from the patient’s lips [16,17]. Several tasks were included such as reading 25 recorded words (word intelligibility calculated as the percentage of words correctly spelled by the patient) [16,17]; spontaneous speech for one minute; sustained production of the phoneme /a/ for as long as possible, performed three times (duration [s], intensity [dB]); counting from 1 to 20 (speech rate [syllables/second]); oral diadochokinesis, during which patients were asked to produce the syllables /pa/, /ta/, /ka/, and the pseudoword /pataka/ as fast as they could with habitual pitch and loudness (parameters estimated; irregular rhythm [presence of absence]; uncontrolled acceleration [presence of absence]). These parameters were selected according to recent guidelines [27] because they represent the acoustic characteristics involved in hypokinetic dysarthria [28,29]. The people conducting the perceptual–acoustic analysis were blinded to the patient’s condition using Praat software [30].

### 2.5. [18F] Flutemetamol PET Study

Each patient underwent [18F] flutemetamol positron emission tomography (PET) with the aim of quantifying cerebral Aβ deposition. PET data have been elaborated with CortexID Suite (GE Healthcare^tm^) software, which allows a fully automated quantification of beta-amyloid brain deposition in different predefined cortical and subcortical regions, and their comparison with normal databases of healthy controls allows an uptake ratio and z-score values to be obtained.

### 2.6. Statistical Analysis

Descriptive statistics were performed for each parameter; continuous variables were expressed as the mean (standard deviation [sd]) and median (range), while frequency and percentages were calculated for categorical variables. The variables were tested for normal distribution using the Kolmogorov–Smirnov test. The primary objective of the study was to find the prevalence of PD patients with a positive [18F] flutemetamol PET study, indicative of brain Aβ deposition, in the long term after surgery. We classified the [18F] flutemetamol PET study as positive if it showed the presence of at least one cortical or subcortical area with a z-score > 2 assessed with the CortexID Suite. The secondary objective of the study was to find the presence of significant differences in clinical and demographic variables between patients with and without brain Aβ deposition. Considering that most variables were not normally distributed, with regard to continuous and ordinal variables, the Mann–Whitney test was used, while for categorical variables, the chi-square independence test was applied. Statistical analyses were performed using IBM SPSS Statistics software for Windows version 20.0 (IBM, Armonk, NY, USA) and Matlab^®^ (Release 2016 b, The MathWorks Inc., Thorofare, NJ, USA). The alpha value was set to 0.05; thus, *p*-values < 0.05 were considered statistically significant.

## 3. Results

From 2012 to 2017, 40 PD patients underwent STN-DBS. Of these, 21 subjects were excluded from the analysis because of missing data (eleven patients), the lack of a PET study (six patients), and the lack of consent to participate (four patients). The remaining 19 PD patients (male: 11; age: 63.52 years; on-stimulation/on-medication UPDRS-III: 17.05) with a mean five-year postoperative follow-up were included. A genetic assessment revealed a heterozygous mutation in the GBA1 gene in three patients. Table 1 shows the demographic and clinical characteristics of the patients, while Table 2 reports motor scores and subscores for the different conditions tested. A detailed description of speech and cognitive variables is reported in Supplementary Table S1.

**Figure 1:**
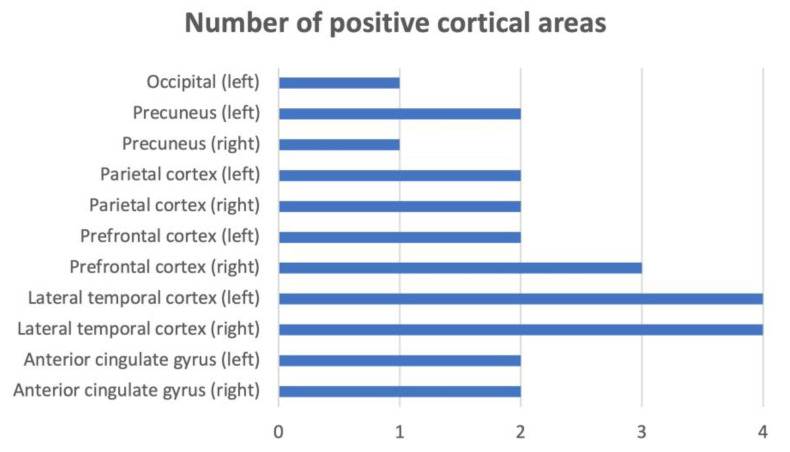
The number of positive cortical areas.

The amyloid-β deposition was found in 21% of patients (4/19), with the prevalent involvement of the prefrontal, limbic, and parietal areas. In particular, both the right and left lateral temporal cortex were involved in the four patients; the right prefrontal cortex was involved in three; the anterior cingulate gyrus, left prefrontal, parietal cortex, and left precuneus were involved in two; and the left occipital and right precuneus were involved in one (see Figure 1).

Compared with patients without amyloid-β deposition, PD patients with a positive 18F-flutemetamol PET study showed a higher preoperative UPDRS-I (*p* = 0.037) score. Table 3 summarizes the main demographic and clinical characteristics of PET-positive and PET-negative cohorts. No differences were found in instrumental speech and gait variables.

## 4. Discussion

In our cohort, a significant proportion of PD patients (i.e., 21%) showed the presence of amyloid-β deposition during the [18F] flutemetamol PET study. This is in line with previous studies, which have reported a 20–30% prevalence of positive amyloid PET scans in patients with PD but without dementia and MCI-PD patients [12,13], suggesting that STN-DBS does not seem to promote amyloid deposition, although a possible selection bias should be taken into account (PD patients with cognitive impairment or severe axial symptoms are usually excluded from surgery). This is an important point because PD patients selected for DBS surgery represent a well-selected subgroup within the entire group of advanced PD patients, thus limiting the generalizability of our results. In particular, one study which included multicenter data from the Parkinson’s Progression Marker Initiative (PPMI) found 10/48 (21%) amyloid-positive PD patients [13] assessed with [18F] florbetaben PET scanning [13]. Interestingly, the increased widespread cortical and subcortical [18F] florbetaben uptake was associated with poorer performance on global cognition (assessed with the Montreal Cognitive Assessment [MOCA]) and impaired performance on the Symbol Digit Modality test (SDMT), which is linked to attention, visual scanning, and motor speed [13]. Another study assessed brain amyloid-β deposition in twenty-five MCI-PD patients through the [18F] flutemetamol PET study, reporting a 32% rate (8/25) of amyloid PET positivity [12]. Even in this case, patients with MCI-PD amyloid-β + showed worse performance in the overall executive domain but not for motor abilities compared to amyloid-negative patients. This result was also found in our cohort. However, it is important to keep in mind that the previously mentioned overall prevalence of PET-positive scans in PD patients (20–30%) was in the range of the literature age-matched control population, suggesting that the possible causative role of PD in amyloid-β deposition needs further confirmation in larger samples [31]. This does not detract from the fact that amyloid-β deposition can negatively influence the clinical phenotype in PD patients, particularly regarding axial and cognitive domains. The dysfunction of the cholinergic circuits, due to the diffusion of the neurodegenerative process at the level of the peduncolo-pontine nucleus, thalamus, basal nucleus of Meynert, and associated cortical cholinergic projections, represents one of the main neuropathological substrates of axial disorders in PD [8,11]. Indeed, this cholinergic phenotype could be associated with a PIGD motor phenotype associated with the freezing of gait (FOG) in the ON medication condition and cognitive impairment [32]. This hypothesis was also confirmed by Muller et al., who found a correlation between the presence of the cortical cholinergic denervation evaluated by PET with tracer for acetylcholinesterase ([(11) C] PMP) and the presence of RBD, falls, walking disorders, and cognitive dysfunctions [33]. Furthermore, comparing PD-fallers vs. PD non-fallers, another group found that acetylcholinesterase levels (indirect markers of cholinergic denervation) at the pedunculopontine nucleus and basal nucleus of Meynert (the two main cerebral cholinergic centers) were significantly lower in the fallers subgroup regardless of the degree of dopaminergic denervation [34]. A subsequent link between axial features, the cholinergic system, and amyloid-β was found by Bohnen et al., who analyzed the correlation between cortical cholinergic denervation, beta-amyloid deposition, and FOG. The results of the study conducted on 143 patients showed a direct correlation between the presence of FOG, the degree of cholinergic denervation, and the deposition of beta-amyloid at the cortical level [8]. The frequency of FOG was also higher in subjects who had both cholinopathy and amyloidopathy at the neo-cortical level. Overall, 90% of patients with FOG also had at least one of the two pathological conditions [8]. Different cross-sectional studies have also confirmed the correlation between beta-amyloid and the PIGD motor phenotype [35,36]. Alves et al. found significantly lower cerebrospinal fluid (CSF) levels of Aβ42, Aβ38, Aβ42/40, and Aβ38/40 in patients with the PIGD phenotype compared to the tremor dominant phenotype. Multivariate regression analyses documented a significant association between CSF beta-amyloid levels, the severity of the PIGD motor phenotype, and lower limb bradykinesia [35]. A subsequent prospective study confirmed that low CSF levels of Aβ42 in the early stages of the disease are predictive of the development of dopa-resistant gait disturbances (variability of gait duration and variability of gait length) with a three-year follow-up [37]. However, in our cohort, no differences were found in the instrumentally assessed axial symptoms between the PET-positive and PET-negative cohorts. This is probably due to the small sample size, supporting the need for larger samples of DBS PD patients. On the contrary, in our cohort, PD patients with positive 18F-flutemetamol PET study showed a higher preoperative UPDRS-I score than patients without amyloid-β deposition. This means that PET-positive patients had a higher cognitive, behavioral, and humoral burden before surgery (quantified through the UPDRS-I score). Focusing on the specific cortical and subcortical areas of the brain in which amyloid-β deposition was found, the involvement of lateral temporal cortices was not surprising. Indeed, a previous prospective study, which included a small cohort of healthy controls (n = 6), patients with PD without dementia (n = 16), and eight patients with PDD studied with the [18F]FDDNP PET scan reported that a higher baseline lateral temporal binding was associated with longitudinal worsening in cognitive performances and progression to dementia among subjects classified as PDND at baseline [38]. The involvement of prefrontal parietal and limbic cortices was also reported in a study by Garon et al., in particular with an increased Aβ uptake in the frontal (i.e., middle frontal cortices and pars triangularis) and parietal regions (i.e., paracentral lobule and precuneus), which overall correlated with poor executive performance [12]. We have to keep in mind that our exploratory study has several limitations, including the small sample size, which reduced the statistical power of our results, not allowing for the performance of correlation analyses between [18F] flutemetamol PET Z-scores and clinical/instrumental variables; the variability of the follow-up duration after surgery between the patients; the lack of the preoperative [18F] flutemetamol PET study; and finally the lack of CSF and ApoE data. However, it represents the first report of the prevalence of amyloid-β deposition in PD patients treated with STN-DBS assessed with a deep clinical instrumental approach focused on axial symptoms.

## 5. Conclusions

To conclude, our results suggest that in the long term, after STN-DBS, a significant percentage of PD patients may present brain amyloid-β deposition. Obviously, future studies with larger samples and preoperative amyloid-β deposition assessments are needed to assess both the possible effect of DBS on amyloid-β deposition (protective, worsening, or irrelevant) and the possible role of amyloid-β deposition in the development of axial and cognitive alterations after surgery. If this is confirmed, the assessment of amyloid-β deposition might be included in the preoperative workup to identify, before the surgery, the patients that already have a higher risk of developing cognitive and axial alterations after STN-DBS.

## Figures and Tables

**Table 1:** Demographic and clinical characteristics of patients.

Variable	Total *n* = 19 NO. (%), Median {RANGE}
Preoperative assessment	
Disease duration at surgery (years)	8.00 {5.00–25.00}
Genetic analysis	
Negative	16 (85.00%)
Positive	3 (GBA1; 15.00%)
PD motor subtype	
PIGD	14 (73.70%)
Indeterminate	2 (10.50%)
TD	3 (15.80%)
LEDD (mg)	1045 {200–1898}
Levodopa responsiveness (%)	58.00 {34.00–93.94}
Postoperative evaluation	
Follow-up duration (years)	5 {3–7}
Age (years)	65 {53–74}
Disease duration (years)	13 {8–28}
PD motor subtype	
PIGD	17 (89.4%)
Indeterminate	1 (5.30%)
TD	1 (5.30%)
LEDD (mg)	774.00 {118.00–1460.00}
PD cognitive status	
PD without dementia	9 (58%)
MCI-PD	8 (41.40%)
PDD	2 (10.60%)

Abbreviations: levodopa equivalent daily dose: LEDD; mild cognitive impairment: MCI; postural instability/gait disorders: PIGD; Parkinson’s disease: PD; Parkinson’s disease with dementia: PDD; tremor dominant: TD.

**Table 2:** Motor scores and subscores for the different conditions tested.

Variables	Stimulation/Medications Conditions				
	Median {Range}				
	Off-Medication	On-Medication	On-Stimulation/Off-Medication	Off-Stimulation/Off-Medication	On-Stimulation/On-Medication
UPDRS part-III	34.00 {25.00–62.00}	13.00 {6.00–31.00}	13.97 {13.00–58.00}	49.00 {29.00–73.00}	13.00 {5.00–38.00}
Akinesia subscore	13.00 {7.00–18.00}	4.00 {1.00–12.00}	11.00 {2.00–23.00}	18.00 {8.00–28.00}	6.00 {1.00–17.00}
Tremor subscore	3.00 {0.00–14.00}	0.00 {0.00–9.00}	3.00 {0.00–7.00}	4.00 {0.00–10.00}	0.00 {0.00–4.00}
PIGD subscore	8.00 {3.00–16.00}	2.00 {0.00–7.00}	9.00 {1.00–13.00}	10.00 {1.00–15.00}	5.00 {1.00–12.00}
Hoehn & Yahr	2.50 {2.00–4.00}	2.00 {2.00–2.50}	2.50 {2.00–5.00}	4.00 {2.00–5.00}	2.50 {2.00–4.00}

Abbreviations: postural instability/gait disorders: PIGD; Unified Parkinson’s Disease Rating Scale: UPDRS.

**Table 3:** Demographic and clinical characteristics of PET-positive and PET-negative cohorts.

Variable	PET-Positive (*n* = 4)	PET-Negative (*n* = 15)
Sex		
Male	2 (50%)	11 (58%)
Female	2 (50%)	8 (42%)
Follow-up duration (years)	5.25 [±0.50]; 5 {5–6}	4.46 [±1.50]; 4 {3–7}
Age (years)	62.75 [±4.03]; 63 {58–67}	63.73 [±5.50]; 65 {53–74}
Disease duration (years)	13.75 [±2.21]; 13 {12–17}	15.33 [±5.27]; 13 {8–28}
PD motor subtype		
PIGD	4 (100%)	13 (86.70%)
Indeterminate	0 (0.00%)	1 (6.70%)
TD	0 (0.00%)	1 (6.70%)
Levodopa equivalent daily dose (mg)	707.75 [±123.01] {580.00–819.00}	797.60 [±375.30] {118.00–1460.00}
PD cognitive status		
PD without dementia	1 (25.00%)	8 (53.30%)
MCI-PD	2 (50.00%)	6 (40.00%)
PDD	1 (25.00%)	1 (6.70%)

## Data Availability

The data that support the findings of this study are available on request from the corresponding author. The data are not publicly available due to privacy or ethical restrictions.

## Supplementary Materials

The following supporting information can be downloaded at https://www.mdpi.com/article/10.3390/jpm14121150/s1. Supplementary Table S1: Cetailed Description of Speech and Cognitive Variables.

## References

[1] Bove F., Mulas D., Cavallieri F., Castrioto A., Chabardès S., Meoni S., Schmitt E., Bichon A., Di Stasio E., Kistner A. (2021). Long-Term Outcomes (15 Years) After Subthalamic Nucleus Deep Brain Stimulation in Patients with Parkinson Disease. Neurology.

[2] Zampogna A., Cavallieri F., Bove F., Suppa A., Castrioto A., Meoni S., Pélissier P., Schmitt E., Bichon A., Lhommée E. (2022). Axial Impairment and Falls in Parkinson’s Disease: 15 Years of Subthalamic Deep Brain Stimulation. NPJ Park. Dis..

[3] Cavallieri F., Campanini I., Gessani A., Budriesi C., Fioravanti V., Di Rauso G., Feletti A., Damiano B., Scaltriti S., Guagnano N. (2023). Long-Term Effects of Bilateral Subthalamic Nucleus Deep Brain Stimulation on Gait Disorders in Parkinson’s Disease: A Clinical-Instrumental Study. J. Neurol..

[4] Deuschl G., Antonini A., Costa J., Śmiłowska K., Berg D., Corvol J.-C., Fabbrini G., Ferreira J., Foltynie T., Mir P. (2022). European Academy of Neurology/Movement Disorder Society-European Section Guideline on the Treatment of Parkinson’s Disease: I. Invasive Therapies. Eur. J. Neurol..

[5] Deuschl G., Antonini A., Costa J., Śmiłowska K., Berg D., Corvol J., Fabbrini G., Ferreira J., Foltynie T., Mir P. (2022). European Academy of Neurology/Movement Disorder Society-European Section Guideline on the Treatment of Parkinson’s Disease: I. Invasive Therapies. Mov. Disord..

[6] Cantiniaux S., Vaugoyeau M., Robert D., Horrelou-Pitek C., Mancini J., Witjas T., Azulay J.-P. (2010). Comparative Analysis of Gait and Speech in Parkinson’s Disease: Hypokinetic or Dysrhythmic Disorders?. J. Neurol. Neurosurg. Psychiatry.

[7] Bohnen N.I., Yarnall A.J., Weil R.S., Moro E., Moehle M.S., Borghammer P., Bedard M.-A., Albin R.L. (2022). Cholinergic System Changes in Parkinson’s Disease: Emerging Therapeutic Approaches. Lancet Neurol..

[8] Bohnen N.I., Frey K.A., Studenski S., Kotagal V., Koeppe R.A., Constantine G.M., Scott P.J.H., Albin R.L., Müller M.L.T.M. (2014). Extra-nigral Pathological Conditions Are Common in Parkinson’s Disease with Freezing of Gait: An in Vivo Positron Emission Tomography Study. Mov. Disord..

[9] Sauerbier A., Jenner P., Todorova A., Chaudhuri K.R. (2016). Non Motor Subtypes and Parkinson’s Disease. Park. Relat. Disord..

[10] Müller M.L.T.M., Frey K.A., Petrou M., Kotagal V., Koeppe R.A., Albin R.L., Bohnen N.I. (2013). Β-amyloid and Postural Instability and Gait Difficulty in Parkinson’s Disease at Risk for Dementia. Mov. Disord..

[11] Lim E.W., Aarsland D., Ffytche D., Taddei R.N., Van Wamelen D.J., Wan Y.-M., Tan E.K., Ray Chaudhuri K., Kings Parcog groupMDS Nonmotor study group (2019). Amyloid-β and Parkinson’s Disease. J. Neurol..

[12] Garon M., Weis L., Fiorenzato E., Pistonesi F., Cagnin A., Bertoldo A., Anglani M., Cecchin D., Antonini A., Biundo R. (2022). Quantification of Brain β-Amyloid Load in Parkinson’s Disease with Mild Cognitive Impairment: A PET/MRI Study. Front. Neurol..

[13] Fiorenzato E., Biundo R., Cecchin D., Frigo A.C., Kim J., Weis L., Strafella A.P., Antonini A. (2018). Brain Amyloid Contribution to Cognitive Dysfunction in Early-Stage Parkinson’s Disease: The PPMI Dataset. J. Alzheimer's Dis..

[14] Reichmann H. (2010). Clinical Criteria for the Diagnosis of Parkinson’s Disease. Neurodegener. Dis..

[15] World Medical Association (2013). World Medical Association Declaration of Helsinki: Ethical Principles for Medical Research Involving Human Subjects. JAMA.

[16] Cavallieri F., Gessani A., Merlo A., Campanini I., Budriesi C., Fioravanti V., Di Rauso G., Feletti A., Damiano B., Scaltriti S. (2023). Interplay between Speech and Gait Variables in Parkinson’s Disease Patients Treated with Subthalamic Nucleus Deep Brain Stimulation: A Long-term Instrumental Assessment. Eur. J. Neurol..

[17] Gessani A., Cavallieri F., Fioravanti V., Campanini I., Merlo A., Di Rauso G., Damiano B., Scaltriti S., Bardi E., Corni M.G. (2023). Long-Term Effects of Subthalamic Nucleus Deep Brain Stimulation on Speech in Parkinson’s Disease. Sci. Rep..

[18] Stebbins G.T., Goetz C.G., Burn D.J., Jankovic J., Khoo T.K., Tilley B.C. (2013). How to Identify Tremor Dominant and Postural Instability/Gait Difficulty Groups with the Movement Disorder Society Unified Parkinson’s Disease Rating Scale: Comparison with the Unified Parkinson’s Disease Rating Scale. Mov. Disord..

[19] Di Rauso G., Cavallieri F., Campanini I., Gessani A., Fioravanti V., Feletti A., Damiano B., Scaltriti S., Bardi E., Corni M.G. (2022). Freezing of Gait in Parkinson’s Disease Patients Treated with Bilateral Subthalamic Nucleus Deep Brain Stimulation: A Long-Term Overview. Biomedicines.

[20] Emre M., Aarsland D., Brown R., Burn D.J., Duyckaerts C., Mizuno Y., Broe G.A., Cummings J., Dickson D.W., Gauthier S. (2007). Clinical Diagnostic Criteria for Dementia Associated with Parkinson’s Disease. Mov. Disord..

[21] Litvan I., Goldman J.G., Tröster A.I., Schmand B.A., Weintraub D., Petersen R.C., Mollenhauer B., Adler C.H., Marder K., Williams-Gray C.H. (2012). Diagnostic Criteria for Mild Cognitive Impairment in Parkinson’s Disease: Movement Disorder Society Task Force Guidelines. Mov. Disord..

[22] Skrahina V., Gaber H., Vollstedt E., Förster T.M., Usnich T., Curado F., Brüggemann N., Paul J., Bogdanovic X., Zülbahar S. (2021). The Rostock International Parkinson’s Disease (ROPAD) Study: Protocol and Initial Findings. Mov. Disord..

[23] Westenberger A., Skrahina V., Usnich T., Beetz C., Vollstedt E.-J., Laabs B.-H., Paul J.J., Curado F., Skobalj S., Gaber H. (2024). Relevance of Genetic Testing in the Gene-Targeted Trial Era: The Rostock Parkinson’s Disease Study. Brain.

[24] Tomlinson C.L., Stowe R., Patel S., Rick C., Gray R., Clarke C.E. (2010). Systematic Review of Levodopa Dose Equivalency Reporting in Parkinson’s Disease. Mov. Disord..

[25] Palmerini L., Mellone S., Avanzolini G., Valzania F., Chiari L. (2013). Quantification of Motor Impairment in Parkinson’s Disease Using an Instrumented Timed up and Go Test. IEEE Trans. Neural Syst. Rehabil. Eng..

[26] Zampieri C., Salarian A., Carlson-Kuhta P., Aminian K., Nutt J.G., Horak F.B. (2010). The Instrumented Timed up and Go Test: Potential Outcome Measure for Disease Modifying Therapies in Parkinson’s Disease. J. Neurol. Neurosurg. Psychiatry.

[27] Rusz J., Tykalova T., Ramig L.O., Tripoliti E. (2021). Guidelines for Speech Recording and Acoustic Analyses in Dysarthrias of Movement Disorders. Mov. Disord..

[28] Skodda S., Visser W., Schlegel U. (2010). Short- and Long-Term Dopaminergic Effects on Dysarthria in Early Parkinson’s Disease. J. Neural. Transm..

[29] Rusz J., Bonnet C., Klempíř J., Tykalová T., Baborová E., Novotný M., Rulseh A., Růžička E. (2015). Speech Disorders Reflect Differing Pathophysiology in Parkinson’s Disease, Progressive Supranuclear Palsy and Multiple System Atrophy. J. Neurol..

[30] Boersma P., Weenink D. (2013). Praat: Doing Phonetics by Computer [Computer Program], Version 5.3.51. https://www.scirp.org/reference/referencespapers?referenceid=830169.

[31] Roberts R.O., Aakre J.A., Kremers W.K., Vassilaki M., Knopman D.S., Mielke M.M., Alhurani R., Geda Y.E., Machulda M.M., Coloma P. (2018). Prevalence and Outcomes of Amyloid Positivity Among Persons Without Dementia in a Longitudinal, Population-Based Setting. JAMA Neurol..

[32] Titova N., Chaudhuri K.R. (2017). Nonmotor Parkinson’s and Future Directions. International Review of Neurobiology.

[33] Müller M.L.T.M., Bohnen N.I., Kotagal V., Scott P.J.H., Koeppe R.A., Frey K.A., Albin R.L. (2015). Clinical Markers for Identifying Cholinergic Deficits in Parkinson’s Disease. Mov. Disord..

[34] Perez-Lloret S., Barrantes F.J. (2016). Deficits in Cholinergic Neurotransmission and Their Clinical Correlates in Parkinson’s Disease. NPJ Parkinson’s Dis..

[35] Alves G., Pedersen K.F., Bloem B.R., Blennow K., Zetterberg H., Borm G.F., Dalaker T.O., Beyer M.K., Aarsland D., Andreasson U. (2013). Cerebrospinal Fluid Amyloid-Beta and Phenotypic Heterogeneity in de Novo Parkinson’s Disease. J. Neurol. Neurosurg. Psychiatry.

[36] Kang J.-H. (2013). Association of Cerebrospinal Fluid β-Amyloid 1-42, T-Tau, P-Tau_181_, and α-Synuclein Levels with Clinical Features of Drug-Naive Patients With Early Parkinson Disease. JAMA Neurol..

[37] Rochester L., Galna B., Lord S., Yarnall A.J., Morris R., Duncan G., Khoo T.K., Mollenhauer B., Burn D.J. (2017). Decrease in Aβ42 Predicts Dopa-Resistant Gait Progression in Early Parkinson Disease. Neurology.

[38] Buongiorno M., Antonelli F., Compta Y., Fernandez Y., Pavia J., Lomeña F., Ríos J., Ramírez I., García J.R., Soler M. (2017). Cross-Sectional and Longitudinal Cognitive Correlates of FDDNP PET and CSF Amyloid-β and Tau in Parkinson’s Disease. J. Alzheimer’s Dis..

