# Supplementary material for: Cerebral Amyloid-β Deposition, Axial Features, and Cognitive Alterations in Patients with Parkinson’s Disease Treated with Bilateral STN-DBS: A Long-Term Cohort Study
Source: J Pers Med. 2024 Dec 10;14(12):1150. doi: 10.3390/jpm14121150 (PMC11676129; doi:10.3390/jpm14121150)
Supplement: Supplementary file 1 [file jpm-14-01150-s001.zip › jpm-3287053-supplementary.pdf]

**Table S1.** Detailed description of speech and cognitive variables.

| Variable | NO. (%); Mean [±SD]; Median {Range} | | | | |
|---|---|---|---|---|---|
| | Preoperative Assessment | | Postoperative Assessment | | |
| | **Off-medication** | **On-medication** | **On-stimulation/off-medication** | **Off-stimulation/off-medication** | **On-stimulation/on-medication** |
| **Speech Variables** | | | | | |
| Speech intelligibility (%) | 89.60 [± 14.38]; 96.00 {64.00-98.00} | 88.60 [± 14.92]; 94.00 {62.00-97.00} | 85.00 [± 19.18]; 94.00 {56.00-100.00} | 76.30 [± 24.45]; 87.50 {48.00-100.00} | 76.40 [± 28.43]; 86.00 {28.00-100.00} |
| Mean intensity of spontaneous speech (dB) | 66.40 [± 2.40]; 67.00 {63.00-69.00} | 64.00 [± 11.31]; 70.00 {49.00-75.00} | 62.20 [± 7.79]; 59.00 {57.00-76.00} | 60.00 [± 5.95]; 57.00 {56.00-70.00} | 61.00 [± 7.74]; 58.00 {55.00-74.00} |
| F0 SD of spontaneous speech (Hz) | 37.56 [± 29.63]; 25.65 {15.26-89.00} | 42.28 [± 22.93]; 32.19 {22.10-76.00} | 45.71 [± 24.32]; 34.93 {24.42-80.24} | 30.19 [± 3.22]; 29.49 {27.03-35.39} | 34.10 [± 10.87]; 29.26 {22.17-42.27} |
| Maximum phonation time (MPT) (seconds) | 16.60 [± 7.26]; 14.00 {8.00-26.00} | 15.40 [± 7.50]; 15.00 {7.00-26.00} | 15.80 [±9 62.]; 13.00 {7.00-32.00} | 11.90 [± 9.23]; 7.00 {5.00-27.00} | 14.40 [± 6.34]; 12.00 {7.00-22.00} |
| Mean intensity of sustained phonation (dB) | 73.20 [± 4.96]; 72.00 {67.00-80.00} | 69.40 [± 7.50]; 71.00 {59.00-78.00} | 65.50 [± 13.55]; 64.00 {52.00-88.00} | 66.40 [± 10.83]; 63.00 {58.00-85.00} | 65.80 [± 15.69]; 62.00 {46.00-88.00} |
| Count rate (sill/sec) | 4.47 [± .78]; 4.30 {3.52-5.67} | 5.09 [± .84]; 5.67 {3.80-5.67} | 4.09 [± 1.34]; 3.60 {2.68-6.14} | 3.91 [± .88]; 3.92 {2.68-5.10} | 4.64 [± 1.96]; 4.64 {2.22-7.28} |
| **Cognitive Variables** | | | | | |
| Phonemic fluency | 32.00 [± 2.86]; 32.61 {28.86-35.35} | | | 28.64 [± 8.14]; 30.90 {15.32-35.79} | |
| Spatial perception localization of numbers | 9.20 [± .83]; 9.00 {8.00-10.00} | | | 7.80 [± 1.30]; 7.00 {7.00-10.00} | |
| 1947 colored Raven's progressive matrices | 26.97 [± 5.10]; 29.48 {21.00-31.98} | | | 25.37 [± 4.70]; 22.76 {21.22-32.20} | |
| Stroop test "time" | 24.35 [± 13.19]; 21.00 {12.50-47.00} | | | 36.95 [± 19.04]; 36.00 {11.50-63.00} | |
| Stroop test "errors" | .80 [± .73]; .75 {.00-2.00} | | | 4.75 [± 5.84]; 1.75 {.00-13.50} | |
| Trail making test part B | 105.80 [± 94.12]; 65.00 {57.00-274.00} | | | 190.20 [± 191.50]; 120.00 {74.00-531.00} | |
